# Multi-omics analysis reveals maternal gut microbiota-derived short-chain fatty acids and progesterone are associated with offspring birth weight in sows

**DOI:** 10.3389/fmicb.2026.1781673

**Published:** 2026-03-10

**Authors:** Xiaojian Xu, Yuwen Chen, Qianhong Ye, Baoyang Xu, Xianghua Yan

**Affiliations:** 1 National Key Laboratory of Agricultural Microbiology, Frontiers Science Center for Animal Breeding and Sustainable Production, Hubei Hongshan Laboratory, College of Animal Sciences and Technology, Huazhong Agricultural University, Wuhan, Hubei, China; 2 College of Animal Science and Technology, College of Veterinary Medicine, Zhejiang A&F University, Hangzhou, Zhejiang, China; 3 National Engineering Research Center for Green Feed and Healthy Breeding, Key Laboratory of Animal Molecular Nutrition, Ministry of Education, Key Laboratory of Animal Nutrition and Feed Science (Eastern of China), Ministry of Agriculture and Rural Affairs, Zhejiang Key Laboratory of Nutrition and Breeding for High-quality Animal Products Institute of Feed Science, College of Animal Science, Zhejiang University, Hangzhou, Zhejiang, China

**Keywords:** fecal metabolomics, gut microbiome, piglet birth weight, short-chain fatty acids, sow, steroid hormone metabolism

## Abstract

**Introduction:**

Piglet birth weight is a key determinant of preweaning survival and subsequent growth performance, yet the role of maternal gut microbiota in relation to offspring birth weight in sows remains incompletely characterized. This study aimed to investigate the association between maternal gut microbiota in late gestation and offspring birth weight in sows.

**Methods:**

Fecal samples were collected from 260 Landrace × Yorkshire (LY) sows at gestation day 100, and sows were categorized into high birth weight (HBW; 1.41 ± 0.02 kg, 16.25 ± 0.25 piglets/litter, *n* = 59) and low birth weight (LBW; 1.07 ± 0.02 kg, 12.19 ± 0.22 piglets/litter, *n* = 52) groups based on the average birth weight of live-born piglets and live litter size. We performed 16S rRNA gene amplicon sequencing and fecal untargeted metabolomics, and quantified fecal short-chain fatty acids (SCFAs) and sex hormones.

**Results:**

Compared with LBW sows, HBW sows showed distinct bacterial community profiles with higher relative abundances of multiple taxa linked to SCFAs production, including *Ruminococcus*, *Oscillibacter*, *Parabacteroides*, and *Bacteroides* (*p* < 0.05). Untargeted metabolomics revealed a clear separation between groups and enrichment of pathways related to primary bile acid biosynthesis and steroid hormone biosynthesis in HBW sows (*p* < 0.05). Consistently, fecal acetate (*p* = 0.005), propionate (*p* = 0.034), isobutyrate (*p* = 0.007), valerate (*p* = 0.036), as well as progesterone (*p* = 0.016), were significantly higher in HBW sows, and these indices were also positively correlated with piglet birth weight. Spearman correlation analysis showed that gut bacterial taxa enriched in the HBW group were positively associated with primary bile acids and sex hormone-related metabolites, which were also positively correlated with piglet birth weight.

**Discussion:**

In conclusion, these multi-omics data indicate that higher piglet birth weight is associated with an SCFAs-enriched gut microbial ecosystem accompanied by enhanced bile acid and steroid hormone-related fecal metabolic profiles during late gestation.

## 1 Introduction

In swine production, litter size and piglet birth weight are key determinants of productivity and profitability (Boonkum et al., 2025). However, genetic selection for increased litter size has been associated with lower mean birth weight and greater within-litter birth weight variation (Quesnel et al., 2008; Langendijk et al., 2023). Low birth weight piglets exhibit delayed and insufficient colostrum intake compared with their normal birth weight counterparts (Declerck et al., 2017), and exhibit impaired intestinal digestive and barrier function (Fang et al., 2024), reduced growth performance (Ruggeri et al., 2024), disrupted follicular development (Almeida et al., 2017), aberrant gut microbiota composition (Li et al., 2018), lower lean meat percentage and compromised meat quality (Gondret et al., 2006), and decreased pre-weaning survival (Mbuthia et al., 2025). Importantly, fetal development depends on maternal nutrient supply via the utero placental circulation throughout gestation, with approximately 60% of fetal weight gain occurring during late gestation (Mcpherson et al., 2004; Kim et al., 2013). Consistent with this, the heritability of piglet birth weight is low (Alves et al., 2018), and nutritional interventions during gestation have been shown to significantly improve piglet birth weight and neonatal outcomes (Langendijk et al., 2023).

The maternal gut microbiome has been increasingly recognized as a potential regulator of offspring birth weight (Chen Y. et al., 2023; Pronovost et al., 2023; Coskun et al., 2025). In humans, maternal gut microbiota composition has been reported to differ between fetal growth restriction (FGR) and uncomplicated pregnancies; for instance, *Bacteroides*, *Faecalibacterium*, and *Lachnospira* have been reported as enriched in women with FGR pregnancies (Tu et al., 2022). In mice, the maternal microbiota is indispensable for placental vascularization and fetal growth; its absence leads to placental insufficiency and decreased fetal weight (Pronovost et al., 2023; Coskun et al., 2025). In sows, accumulating evidence indicates that dietary supplementation with prebiotics or probiotics increases piglet birth weight (Han et al., 2022; Li et al., 2022; Huang et al., 2023; Ma et al., 2023; Li S. et al., 2024). These benefits may be mediated by modulation of gut microbiota composition, improved placental function and steroid hormone synthesis, and increased intestinal levels of short-chain fatty acids (SCFAs). Previous studies reported that probiotic supplementation starting on gestation day 85 increased piglet birth weight and reduced the proportion of weak piglets; these effects were associated with activation of placental mechanistic target of rapamycin (mTOR) signaling, upregulation of placental glucose and amino-acid transporters, and modulation of the maternal gut microbiota (Li S. et al., 2024). Studies have shown that dietary supplementation with sodium butyrate and sodium acetate during sow gestation increases piglet birth weight and reduces the proportion of weak piglets (birth weight < 0.80 kg), respectively (Zhao et al., 2021; Qi et al., 2023). Conversely, an increased abundance of potentially pathogenic bacteria (e.g., *Bacteroides fragilis*) and other lipopolysaccharide-producing taxa has been associated with pro-inflammatory responses and adverse reproductive outcomes, including stillbirth (Chen et al., 2022). Despite these advances, the gut microbial taxa and functions linked to piglet birth weight remain unclear, and maternal gut microbiome composition and fecal metabolite profiles in sows producing piglets with high versus low piglet birth weights are still insufficiently characterized.

To address this gap, we collected fecal samples from 260 Landrace × Yorkshire (LY) sows at gestation day 100 and recorded the birth weights of their offspring. Sows were classified into high birth weight (HBW; 1.41 ± 0.02 kg, 16.25 ± 0.25 piglets/litter, *n* = 59) and low birth weight (LBW; 1.07 ± 0.02 kg, 12.19 ± 0.22 piglets/litter, *n* = 52) groups according to average piglet birth weight and live litter size. We then profiled and compared the maternal gut microbiota composition and fecal metabolomic features between the two groups. By integrating gut microbiome and metabolome profiling, this study aims to (1) characterize between-group differences in maternal gut microbial community and metabolic profiles, and (2) identify specific microbial taxa and metabolites associated with piglet birth weight, thereby providing a reference for developing targeted nutritional and microbiome-based strategies to improve birth weight in commercial swine production.

## 2 Materials and methods

### 2.1 Experimental design and animals

This study was conducted on a commercial farm in Huanggang, China, and included LY sows with parity 2–4 that were managed under the same housing and feeding conditions. Sows were fed a gestation diet formulated to meet NRC (2012) nutrient requirements (Table 1). During gestation, sows were fed twice daily at 07:00 and 14:30, with a standardized feeding allowance of 2.30 kg/day from breeding to gestation day 85 and 3.50 kg/day from day 86 to 114. On gestation day 100 (a period of rapid fetal weight gain), fresh fecal samples were collected in the morning from all enrolled sows and stored at −80 °C until analysis. We initially collected fecal samples from 293 pregnant sows. After farrowing, we reviewed farrowing records and reproductive outcomes and excluded sows with dystocia, preterm or prolonged farrowing, and low prolificacy (live litter size < 9). After these predefined exclusions, 260 sows had complete and qualified reproductive records and formed the final eligible cohort. The farrowing process was monitored, and live litter size, the average birth weight of live-born piglets, and the numbers of stillborn and mummified piglets were recorded. For between-group comparisons, we used an extreme-phenotype design based on litter live birth weight (which reflects both live litter size and average piglet birth weight). Within the 260-sow cohort, sows were ranked by litter live birth weight. We then selected sows from the top and bottom of this ranking and defined two groups: high birth weight (HBW; 1.41 ± 0.02 kg, 16.25 ± 0.25 piglets/litter, *n* = 59) and low birth weight (LBW; 1.07 ± 0.02 kg, 12.19 ± 0.22 piglets/litter, *n* = 52). This extreme-phenotype design increases biological contrast and reduces phenotype overlap, which helps detect microbiome-associated differences. All fecal-based assays (16S rRNA gene amplicon sequencing, untargeted metabolomics, SCFA quantification, and fecal hormone measurements) were performed on the same 111 sows. No antibiotics were administered during the sampling period.

**Table 1:** Ingredient composition and nutritional levels of the basal diet (as-fed-basis).

Item	Content, %
Corn	34.00
Wheat	15.00
Rice bran meal	12.00
Wheat bran	23.00
Soybean meal (43%)	6.00
Soybean oil	1.00
Fermentation material	5.00
NaCl	0.40
Limestone	1.00
Choline chloride (50%)	0.12
Dicalcium phosphate	1.30
L-Lys HCl (98%)	0.16
DL-Methionine (98%)	0.04
L-Threonine (98.5%)	0.05
Phytase	0.02
Vitamin-mineral premix^1^	0.91
Total	100.00
Nutrient levels^2^, %	
Digestible energy, Mcal/kg	2.97
Crude protein	13.60
Calcium	0.72
Total phosphorus	0.79
Total Lys	0.92
Total Met + Cys	0.64

1 The premix provided the following per kilogram of complete diets: Zn 120 mg; Cu 15 mg; Fe 120 mg; Mn 60 mg; Cr 0.1 mg; I 1.2 mg; Se 0.3 mg; vitamin A 10,000 IU; vitamin D3 1,800 IU; vitamin E 100 IU; vitamin K3 4.5 mg; vitamin B1 2 mg; vitamin B2 8 mg; vitamin B6 4 mg; vitamin B12 30 mcg; niacin 40 mg; pantothenic acid 30 mg; folic acid 4 mg; biotin 500 mcg.

2 Digestible energy, crude protein, calcium, total phosphorus, total lysine, and total methionine + cysteine were calculated values, which were calculated according to the nutrient concentration of each ingredient multiplied by its proportion in the diet, and the contributions from all ingredients were then summed to obtain the final dietary values. The nutrient concentrations of individual ingredients were based on the tables of feed composition and nutritive values in China (China Feed Database, 2020).

### 2.2 Bacterial 16S rRNA gene amplicon sequencing and data analysis

Fecal samples were homogenized using 6 mm bead-beating beads, and microbial genomic DNA was then isolated with the DNeasy PowerSoil Pro Kit (QIAGEN GmbH, Hilden, North Rhine-Westphalia, Germany) following the manufacturer’s instructions. For amplicon sequencing, the V3–V4 region of the bacterial 16S rRNA gene was amplified as described previously (Ye et al., 2024). Amplicons were purified and quantified prior to library preparation and sequencing. Sequencing libraries were prepared using the NextSeq 1000/2000 P1 Reagent Kit (Illumina Inc., San Diego, CA, United States) and sequenced on an Illumina NextSeq 2000 platform (Illumina Inc., San Diego, CA, United States). After quality control with fastp (v0.19.6), paired-end reads were assembled into merged sequences using FLASH (v1.2.7). Denoising and chimera removal were conducted in QIIME 2 (v2022.2) using the DADA2 plugin, yielding amplicon sequence variants (ASVs). Taxonomy annotation of the ASVs was performed using the Naive Bayes classifier in QIIME 2, with the SILVA database (release 138.1) used for bacterial annotation, applying a confidence threshold of 70%. To standardize sequencing depth across samples, feature tables were subsampled to 21,995 reads per sample for 16S datasets. Downstream statistical analysis and visualization were performed on the Majorbio Cloud platform.^1^ Based on the ASVs information, rarefaction curves and alpha diversity indices were calculated using Mothur (v1.30.1) and visualized in R (v4.2.3). Beta-diversity was calculated in QIIME 2 and visualized in R (v4.2.3). Linear discriminant analysis effect size (LEfSe) was applied to identify differentially abundant taxa between groups (LDA > 2, *p* < 0.05). Spearman correlation analysis was performed to assess the relationships between piglet birth weight and differential gut microbial taxa, as well as between differential gut microbial taxa and differential fecal metabolites related to steroids, bile acids, and associated derivatives.

### 2.3 Untargeted metabolomics and data analysis

Metabolite extraction followed a previously reported protocol (Want et al., 2013). Briefly, 50 mg of fecal sample was extracted with 400 μL methanol:water (4:1, v/v) containing 0.02 mg/mL L-2-chlorophenylalanine, and the supernatant was collected for liquid chromatography-tandem mass spectrometry (LC-MS/MS) analysis. Instrument performance was monitored using a pooled quality control (QC) sample, prepared by combining equal volumes of all samples, which was injected once every 15 runs. Data were acquired on an Ultra-High-Performance Liquid Chromatography (UHPLC) system coupled to a Q Exactive HF-X Orbitrap mass spectrometer (Thermo Fisher Scientific Inc., Waltham, MA, United States). The raw data were processed using Progenesis QI software (Waters Corp., Milford, MA, United States). Metabolites were annotated by matching features to HMDB, Metlin, and the Majorbio database. The resulting feature table was then uploaded to the Majorbio Cloud platform (see text footnote 1) for analyses. Multivariate discrimination between groups was assessed using orthogonal partial least squares discriminant analysis (OPLS-DA). Model quality was evaluated by cross-validation (reported as *R*^2^ and *Q*^2^) and permutation testing to assess the risk of overfitting. Metabolites contributing to group separation were ranked by variable importance in projection (VIP). For univariate comparisons, metabolite abundances were first log10-transformed, followed by two-sided unpaired *t*-tests between groups. Multiple-testing correction was implemented with the Benjamini–Hochberg procedure to control the false discovery rate (FDR). Metabolites were considered differential if they met both thresholds: FDR < 0.05 and VIP > 1. Differential metabolites were then mapped to pathways using Kyoto Encyclopedia of Genes and Genomes (KEGG) database. Spearman correlation analysis was performed to assess the associations between piglet birth weight and differential fecal metabolites related to steroids, bile acids, and associated derivatives. Additionally, MetOrigin (Yu G. et al., 2024) was employed to integrate statistical correlations and biological pathways between the gut microbiome and metabolome.

### 2.4 Measurement of fecal SCFAs

Fecal levels of SCFAs were measured by gas chromatography as previously described (Xu et al., 2021). Briefly, 1 g of fecal sample was homogenized with 1 mL methanol, and the supernatant was mixed with 25% metaphosphoric acid at a 4:1 (v/v) ratio. After incubation at 4 °C overnight, the supernatant was obtained by centrifugation at 12,000 × g for 10 min at 4 °C and analyzed by gas chromatography (Trace 1300, Thermo Fisher Scientific Inc., Waltham, MA, United States). To quantify individual SCFAs, calibration curves were constructed for each compound over the concentration range of 0–5,000 μg/mL.

### 2.5 Measurement of fecal estradiol and progesterone

Fecal estradiol and progesterone were quantified using the Iodine[^125^I] Estradiol Radioimmunoassay Kit and Iodine[^125^I] Progesterone Radioimmunoassay Kit (Beijing North Biotechnology Institute Co., Ltd., Beijing, China) according to the manufacturer’s instructions. Briefly, 0.2 g of fecal sample was homogenized in 1 mL of 80% ethanol, incubated at 70 °C for 15 min, and centrifuged to collect the supernatant. The residual pellet was subsequently re-extracted with 0.5 mL of 80% methanol, and centrifuged to collect the supernatant. The combined supernatants were evaporated to dryness at 60 °C in a water bath. The dried residue was reconstituted in 1 mL of buffer solution and vortexed thoroughly to ensure homogeneity. According to preliminary experiments, samples were diluted to appropriate concentrations prior to analysis. To quantify estradiol and progesterone, calibration curves were generated over 0–4,000 pg/mL and 0–100 ng/mL, respectively.

### 2.6 Statistical analysis

Statistical analyses and data visualization were performed using SPSS (v27.0), GraphPad Prism (v8.0), and R (v4.2.3). Data were tested for normality within each group using the Shapiro–Wilk test. Piglet birth weight was analyzed using the following general linear model:


$$Y_{i}=\mu +G_{g(i)}+\beta_{1}N_{i}+P_{p(i)}+B_{k(i)}+e_{i},$$

where *Y_i_* is the dependent variable, *μ* is the overall mean, *G*_*g*(*i*)_ is the fixed effect of group, *N_i_* is live litter size, *P*_*p*(*i*)_ is the fixed effect of parity, *B*_*k*(*i*)_ is the fixed effect of sampling batch, and *e_i_* is the residual error. Fecal estradiol and progesterone were analyzed using the same model, where *Y_i_* represents the measured fecal estradiol or progesterone concentration. Group was modeled as the main effect, live litter size was included as a covariate, and parity and batch were included as fixed effects. Parity, live litter size, and the numbers of stillborn and mummified piglets were compared between groups using the Mann–Whitney *U* test. For SCFAs, group comparisons were conducted using a two-sided Student’s *t*-test. Spearman correlation analysis was performed to assess the associations between piglet birth weight and fecal SCFA concentrations, as well as fecal progesterone and estradiol levels. Alpha diversity indices were compared between groups using the Mann–Whitney *U* test. Beta diversity was calculated based on Bray–Curtis distances, and the effect of group was tested using PERMANOVA with parity included as a fixed effect. Genera-level differences between groups were assessed using the Mann–Whitney *U* test. Differential genera were further identified using LEfSe, with an LDA > 2 and *p* < 0.05 as the significance thresholds. Metabolites were considered differential when they met both criteria: FDR < 0.05 and VIP > 1. The data are presented as mean ± SEM. Statistical significance was presented as ^*^p < 0.05, ^**^p < 0.01, and ^***^p < 0.001.

## 3 Results

### 3.1 Gut bacterial community differences associated with piglet birth weight in sows

**Figure 1:**
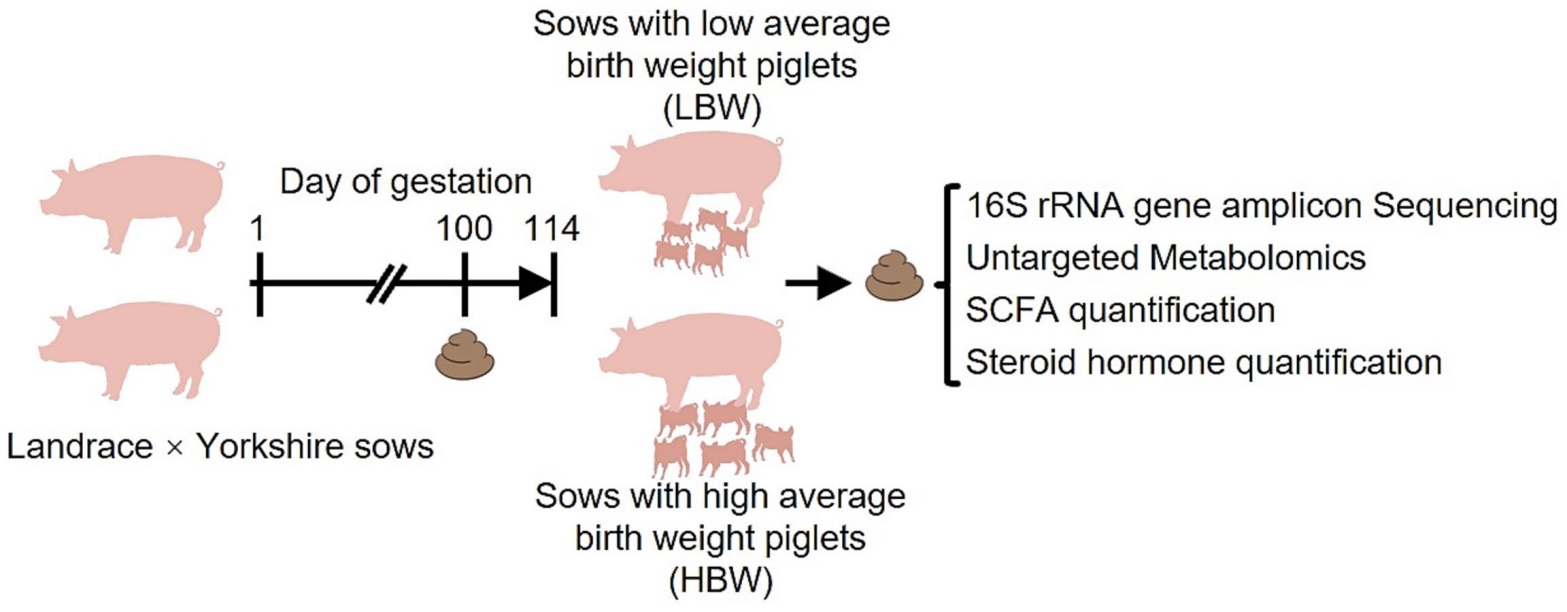
Schematic of the experimental design. Fecal samples were collected from 260 sows on gestation day 100, and birth weights of their offspring were subsequently recorded. Fecal samples from the HBW and LBW groups were analyzed by 16S rRNA gene amplicon sequencing, untargeted metabolomics, SCFA quantification, and steroid hormone quantification to characterize between-group differences in the maternal gut microbiota and metabolic profiles. HBW, high birth weight; LBW, low birth weight; SCFA, short-chain fatty acid.

To test the hypothesis that differences in gut microbiota composition and metabolic profiles were associated with piglet birth weight, fecal samples from the HBW and LBW groups were analyzed using 16S rRNA gene amplicon sequencing, untargeted metabolomics, SCFA quantification, and steroid hormone quantification (Figure 1). The HBW group exhibited a significantly higher live litter size than the LBW group (*p* < 0.001) (Table 2), whereas sow parity did not differ significantly between groups. In a general linear model adjusted for live litter size, parity, and sampling batch, piglet birth weight remained significantly higher in the HBW group than in the LBW group (*p* < 0.001) (Table 2). The numbers of stillborn and mummified piglets did not differ between groups (Table 2), which suggests that overall maternal condition and pregnancy status were comparable between groups.

**Table 2:** Analysis of sow reproductive performance between the HBW and LBW groups^1^.

Item	LBW	HBW	SEM	*p*-value
Live litter size, *n/*litter	12.19^b^	16.25^a^	0.26	<0.001
Piglet birth weight, kg	1.07^b^	1.41^a^	0.02	<0.001
Parity	2.98	2.90	0.08	0.583
Stillborn piglets, *n/*litter	0.37	0.37	0.08	0.719
Mummified piglets, *n/*litter	0.13	0.08	0.04	0.578

SEM, standard error of the mean.

Means within the same row with different superscript letters are significantly different.

1 LBW (low birth weight, *n* = 52); HBW (high birth weight, *n* = 59).

**Figure 2:**
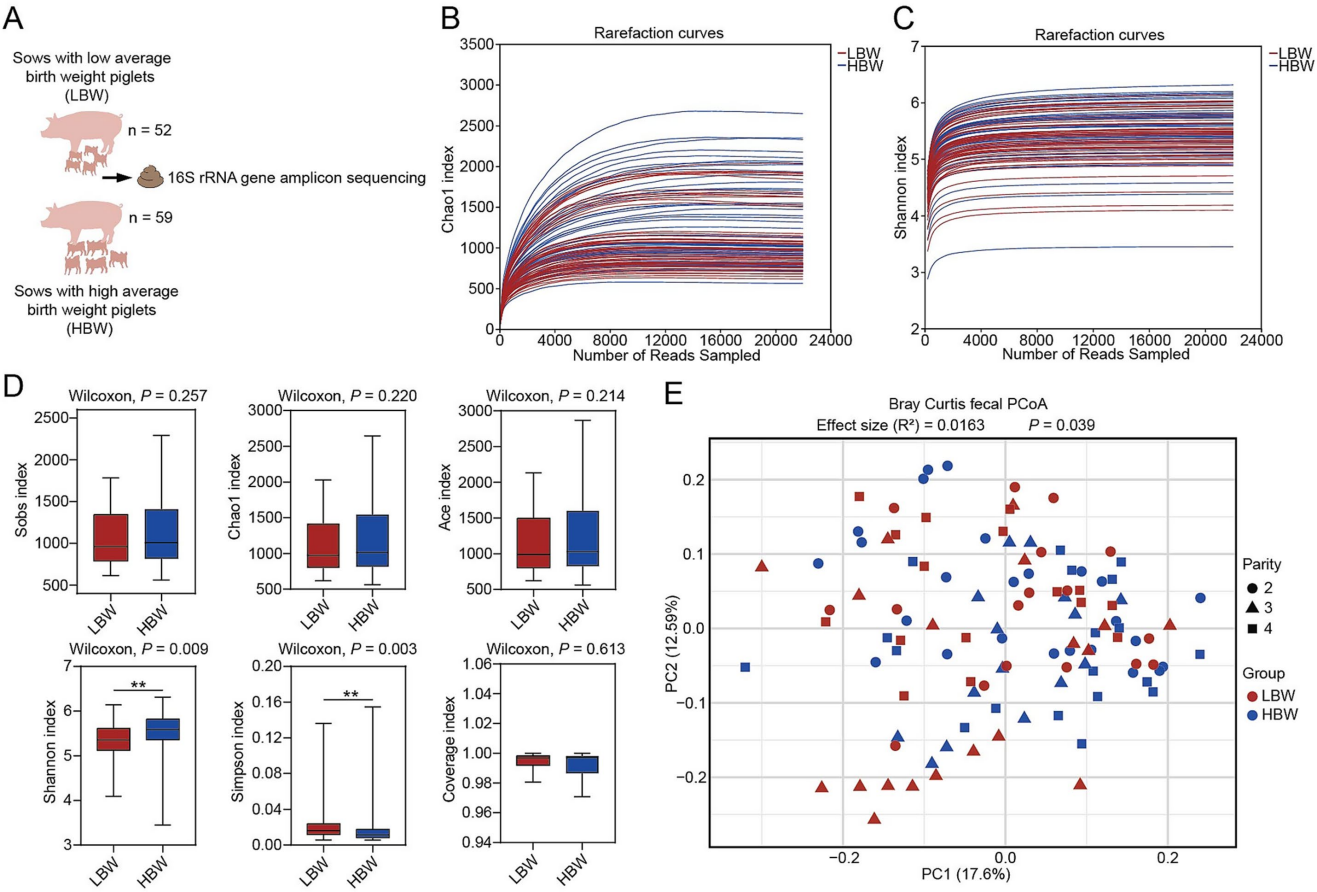
Analysis of gut bacterial diversity between the HBW and LBW groups. **(A)** Schematic of the experimental design. **(B,C)** Rarefaction curves of the Chao1 **(B)** and Shannon **(C)** indices based on 16S rRNA gene amplicon sequencing. **(D)** Analysis of gut bacterial alpha diversity between the HBW and LBW groups, including Sobs, Chao1, ACE, Shannon, Simpson, and Good’s coverage indices. **(E)** Beta diversity was assessed using Bray–Curtis distances, and group differences were tested by PERMANOVA with parity included as a fixed effect. *n* = 52 for LBW group, *n* = 59 for HBW group. HBW, high birth weight; LBW, low birth weight; PCoA, principal coordinates analysis, ^**^p < 0.01.

To profile gut bacterial communities in relation to piglet birth weight, fecal samples from both groups were analyzed by 16S rRNA gene amplicon sequencing (Figure 2A). Rarefaction curves based on the Chao1 and Shannon indices approached a plateau, suggesting that the sequencing depth was sufficient to capture bacterial richness and diversity in the 16S rRNA gene amplicon data (Figures 2B,C). Compared with the LBW group, the HBW group had a significantly higher Shannon index (*p* = 0.009) and a significantly lower Simpson index (*p* = 0.003), while no significant differences were observed in the Sobs, Chao1, ACE, and Good’s Coverage indices (*p* > 0.05) (Figure 2D). Principal coordinates analysis (PCoA) based on Bray–Curtis distances showed a modest separation in gut microbial community structure between the HBW and LBW groups (Figure 2E). This pattern was supported by PERMANOVA, which indicated a significant group effect after adjusting for parity (*R*^2^ = 0.0163, *p* = 0.039) (Figure 2E).

**Figure 3:**
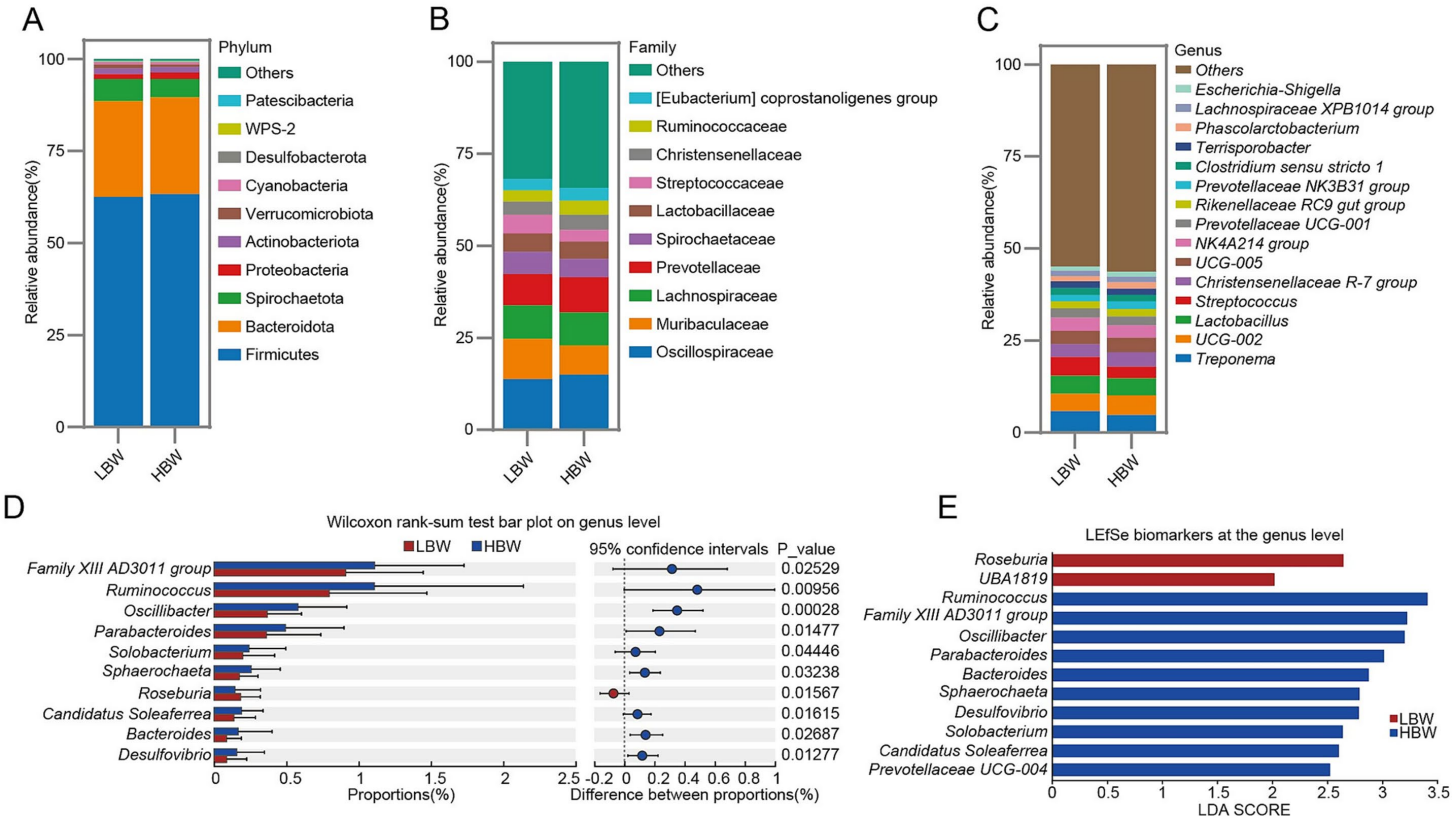
Analysis of gut bacterial communities between the HBW and LBW groups. **(A–C)** Stacked bar charts of gut bacterial taxonomic composition at the phylum **(A)**, family **(B)**, and genus **(C)** levels. **(D)** Differences in gut bacterial communities at the genus level. **(E)** Bar chart of gut bacterial taxa identified by LEfSe analysis at the genus level (LDA > 2, *p* < 0.05). *n* = 52 for LBW group, *n* = 59 for HBW group. HBW, high birth weight; LBW, low birth weight; LEfSe, linear discriminant analysis effect size.

Firmicutes, Bacteroidota, and Spirochaetota were identified as the most abundant bacterial phyla in both the HBW and LBW groups (Figure 3A). At the family level, Oscillospiraceae, Muribaculaceae, Lachnospiraceae, Prevotellaceae, and Spirochaetaceae were predominant in both groups (Figure 3B). The most abundant bacterial genera included *Treponema*, *UCG-002*, *Lactobacillus*, *Streptococcus*, and *Christensenellaceae R-7 group* (Figure 3C). Based on genus-level taxonomic assignments, the HBW group had significantly higher relative abundances of *Family XIII AD3011 group*, *Ruminococcus*, *Oscillibacter*, *Parabacteroides*, *Solobacterium*, *Sphaerochaeta*, *Candidatus Soleaferrea*, *Bacteroides*, and *Desulfovibrio* (*p* < 0.05), and a significantly lower abundance of *Roseburia* (*p* < 0.05) than the LBW group (Figure 3D and Table 3). At the genus level, LEfSe identified *Roseburia* and *UBA1819* as biomarker genera of the LBW group, whereas *Ruminococcus*, *Family XIII AD3011 group*, *Oscillibacter*, *Parabacteroides*, *Bacteroides*, *Sphaerochaeta*, *Desulfovibrio*, *Solobacterium*, *Candidatus Soleaferrea*, and *Prevotellaceae UCG-004* were identified as biomarker genera of the HBW group (Figure 3E).

**Table 3:** Significantly different gut bacterial taxa at the genus and species levels between the HBW and LBW groups (relative abundance >0.05%)^1^.

Bacteria	LBW	HBW	SEM	*p*-value
Genus, %				
*Family XIII AD3011 group*	0.91^b^	1.11^a^	0.06	0.025
*Ruminococcus*	0.80^b^	1.11^a^	0.08	0.010
*Oscillibacter*	0.37^b^	0.58^a^	0.03	<0.001
*Parabacteroides*	0.36^b^	0.49^a^	0.04	0.015
*Solobacterium*	0.20^b^	0.24^a^	0.02	0.045
*Sphaerochaeta*	0.18^b^	0.26^a^	0.02	0.032
*Roseburia*	0.18^b^	0.15^a^	0.02	0.016
*Candidatus Soleaferrea*	0.14^b^	0.19^a^	0.01	0.016
*Bacteroides*	0.09^b^	0.17^a^	0.02	0.027
*Desulfovibrio*	0.09^b^	0.16^a^	0.02	0.013
*Prevotellaceae UCG-004*	0.06^b^	0.09^a^	0.01	0.047
Species, %				
*Porphyromonadaceae bacterium DJF B175*	0.29^b^	0.38^a^	0.03	0.025

SEM, standard error of the mean.

Means within the same row with different superscript letters are significantly different.

1 LBW (low birth weight, *n* = 52); HBW (high birth weight, *n* = 59).

### 3.2 Gut microbial metabolic differences associated with piglet birth weight in sows

**Figure 4:**
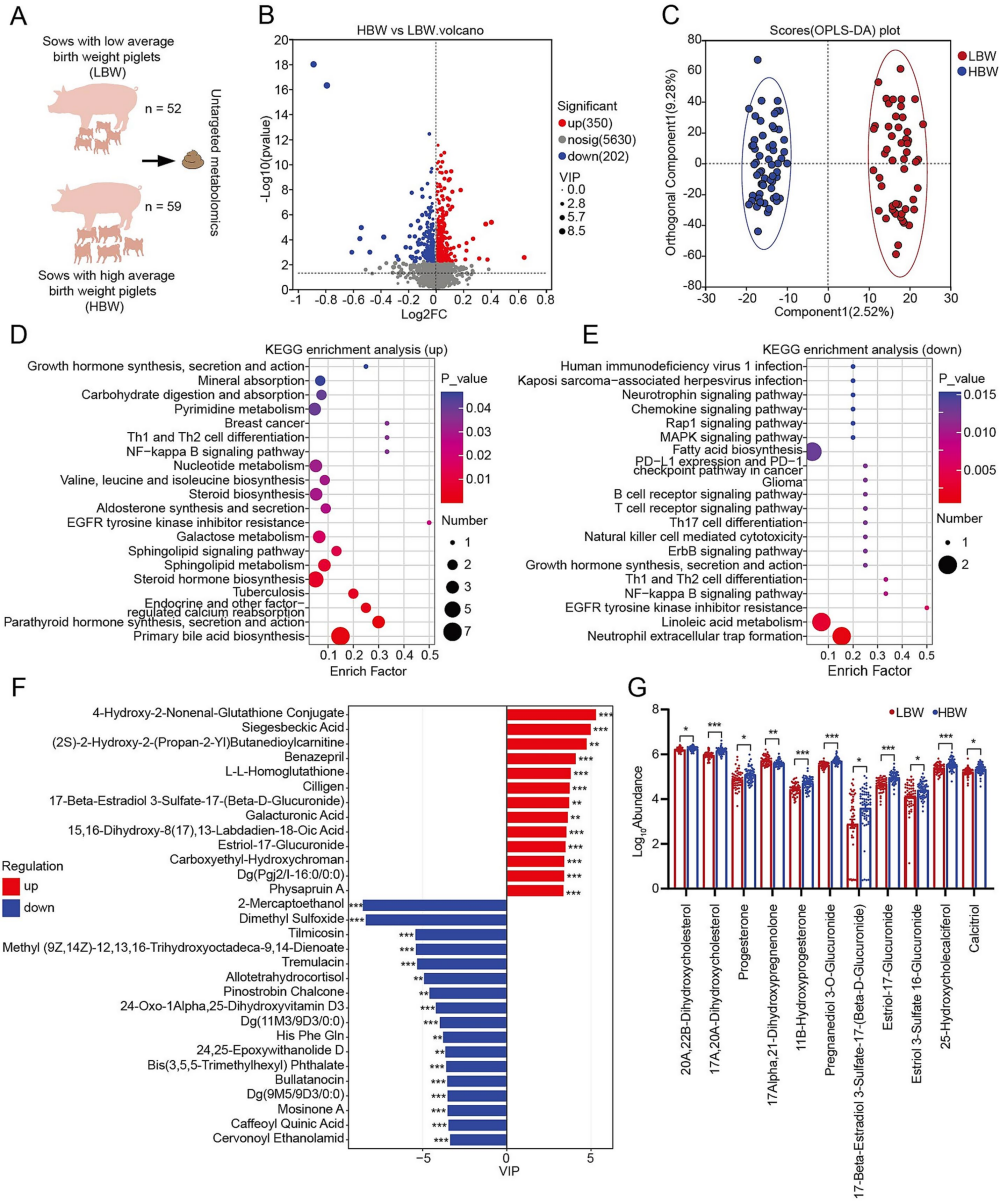
Analysis of fecal metabolomic profiles between the HBW and LBW groups. **(A)** Schematic of the experimental design. **(B)** Volcano plot of differential metabolites in both positive and negative ion modes. **(C)** OPLS-DA analysis of samples based on fecal metabolomic profiles. **(D)** KEGG pathway enrichment analysis of metabolites significantly upregulated in the HBW group than in the LBW group. **(E)** KEGG pathway enrichment analysis of metabolites significantly downregulated in the HBW group than in the LBW group. **(F)** Top 30 significantly differential metabolites ranked by VIP scores from the OPLS-DA model. **(G)** Significantly differential steroid metabolism-related metabolites between the HBW and LBW groups. *n* = 52 for LBW group, *n* = 59 for HBW group. KEGG, Kyoto Encyclopedia of Genes and Genomes; HBW, high birth weight; LBW, low birth weight; OPLS-DA, orthogonal partial least squares discriminant analysis. ^*^p < 0.05, ^**^p < 0.01, and ^***^p < 0.001.

Untargeted metabolomics was performed to characterize the differences in gut metabolic profiles between the HBW and LBW groups (Figure 4A). A total of 6,182 metabolites were detected and could be successfully annotated, of which 350 metabolites in HBW group were significantly higher than that in LBW group, and 202 metabolites in HBW group were significantly lower than that in LBW group (Figure 4B). OPLS-DA analysis revealed that HBW sows exhibited distinct metabolic profiles compared with LBW sows during late gestation (Figure 4C). KEGG pathway enrichment analysis of the 350 metabolites significantly upregulated in the HBW group revealed several key pathways, including primary bile acid biosynthesis, parathyroid hormone synthesis, secretion and action, steroid hormone biosynthesis, and steroid biosynthesis (*p* < 0.05) (Figure 4D). The 202 metabolites that were significantly downregulated in the HBW group were mainly enriched in the neutrophil extracellular trap formation, linoleic acid metabolism, EGFR tyrosine kinase inhibitor resistance, and NF-kappa B signaling pathway (*p* < 0.05) (Figure 4E). Differential metabolites were ranked by VIP scores from the OPLS-DA model, and the top 30 features were further examined (Figure 4F). Among these top-ranked metabolites, 4-hydroxy-2-nonenal-glutathione conjugate, siegesbeckic acid, (2S)-2-hydroxy-2-(propan-2-yl)butanedioylcarnitine, benazepril, and L-L-Homoglutathione were enriched in the HBW group (Figure 4F). Notably, several steroid hormone-related metabolites were significantly higher in HBW sows, such as estriol-17-glucuronide and 17β-estradiol 3-sulfate-17-(β-D-glucuronide) (Figure 4F). Conversely, the LBW group showed significant enrichment of metabolites including 2-Mercaptoethanol, dimethyl sulfoxide, tilmicosin, methyl (9Z,14Z)-12,13,16-trihydroxyoctadeca-9,14-dienoate, and tremulacin (Figure 4F). Additionally, several metabolites involved in steroid-related metabolism, including 20α,22β-dihydroxycholesterol, 17α,20α-dihydroxycholesterol, progesterone, 11β-hydroxyprogesterone, pregnanediol 3-O-glucuronide, estriol-17-glucuronide, and estriol 3-sulfate 16-glucuronide, were significantly higher in the HBW group than in the LBW group (*p* < 0.05), whereas 17α,21-dihydroxypregnenolone was significantly lower in the HBW group than in the LBW group (*p* < 0.05) (Figure 4G).

### 3.3 Differences in fecal hormones and SCFA levels associated with piglet birth weight in sows

**Figure 5:**
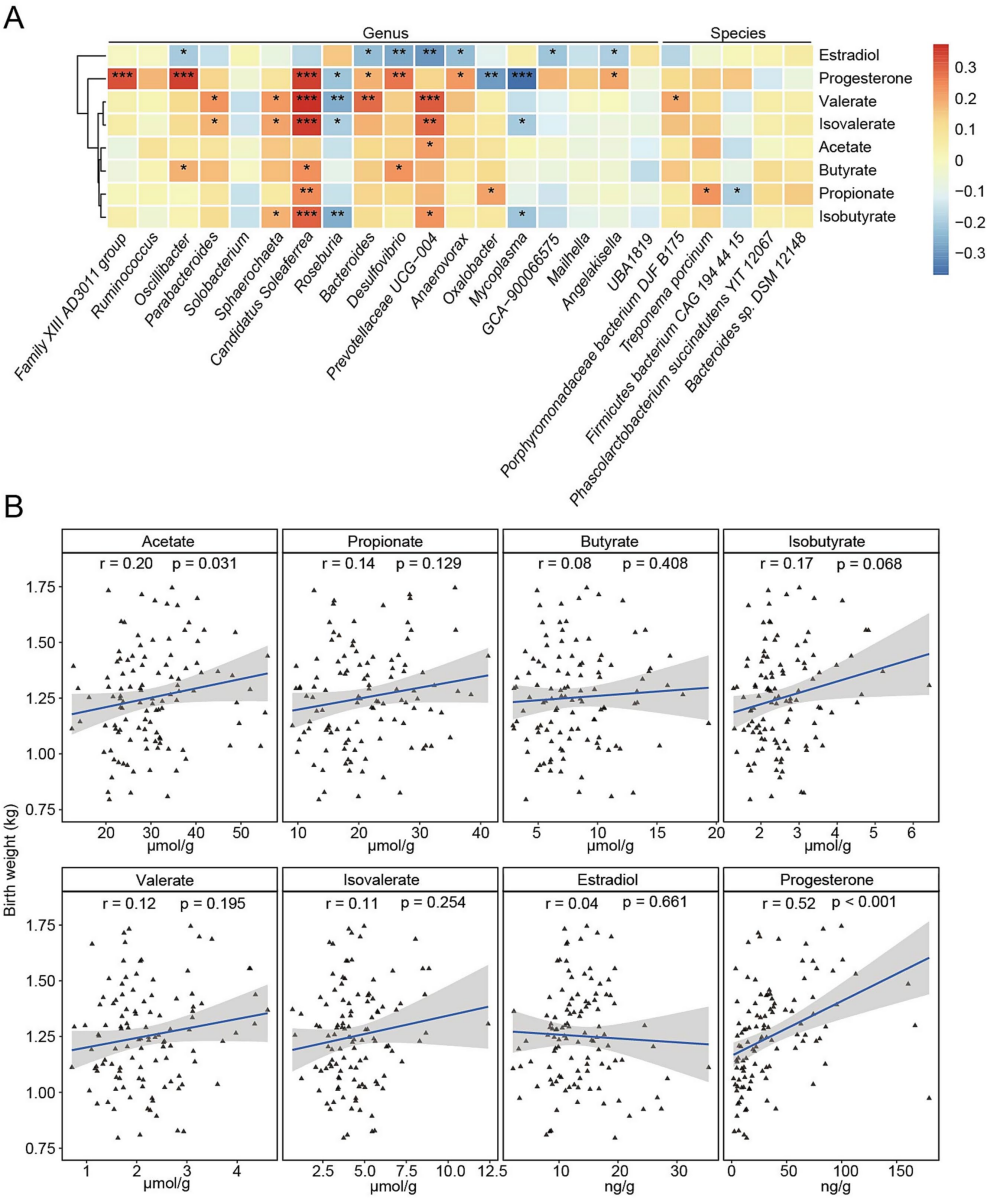
Associations between fecal SCFAs, fecal sex steroid hormones, and piglet birth weight in HBW and LBW sows. **(A)** Spearman correlation heatmap between fecal SCFAs, estradiol, progesterone, and bacterial taxa (genus and species levels) that were significantly different between the HBW and LBW groups. In the figure, the red color represents a positive correlation, while the blue color represents the negative correlation. ^*^p < 0.05, ^**^p < 0.01, and ^***^p < 0.001. **(B)** Spearman correlations between piglet birth weight and fecal SCFAs, estradiol, and progesterone. Each point represents one sow, and the fitted line and shaded area indicate the regression trend and 95% confidence interval.

The HBW group exhibited significantly higher fecal concentrations of acetate (*p* = 0.005), propionate (*p* = 0.034), isobutyrate (*p* = 0.007), and valerate (*p* = 0.036) compared to the LBW group, and isovalerate (*p* = 0.085) showed an increasing trend in the HBW group (Table 4). After adjusting for live litter size, parity, and sampling batch, fecal progesterone concentrations were significantly higher in the HBW group than in the LBW group (*p* = 0.016), whereas fecal estradiol concentrations showed no difference between groups (*p* = 0.526) (Table 4). Spearman correlation analysis showed that taxa enriched in the HBW group displayed a consistent association pattern with fecal steroid hormones and SCFAs (Figure 5A). Specifically, HBW-enriched taxa tended to be negatively correlated with fecal estradiol and positively correlated with fecal progesterone, and these taxa also showed positive correlations with fecal SCFAs (Figure 5A). In contrast, taxa enriched in the LBW group were generally negatively correlated with fecal progesterone and SCFAs (Figure 5A). Among SCFAs, acetate showed a modest but significant positive correlation with piglet birth weight (*r* = 0.20, *p* = 0.031), whereas isobutyrate showed a positive association with a significant trend (*r* = 0.17, *p* = 0.068) (Figure 5B). Propionate (*r* = 0.14, *p* = 0.129), butyrate (*r* = 0.08, *p* = 0.408), valerate (*r* = 0.12, *p* = 0.195), and isovalerate (*r* = 0.11, *p* = 0.254) also showed positive correlation with piglet birth weight, but none were statistically significant. In addition, piglet birth weight was significantly positively correlated with fecal progesterone (*r* = 0.52, *p* < 0.001), whereas fecal estradiol was not significantly associated with piglet birth weight (*r* = 0.04, *p* = 0.661) (Figure 5B).

**Table 4:** Analysis of fecal levels of SCFAs, estradiol, and progesterone between the HBW and LBW groups^1^.

Item	LBW	HBW	SEM	*p*-value
SCFAs, μmol/g				
Acetate	27.69^b^	32.47^a^	0.86	0.005
Propionate	19.46^b^	22.38^a^	0.69	0.034
Butyrate	7.65	8.50	0.32	0.186
Isobutyrate	2.35^b^	2.80^a^	0.08	0.007
Valerate	2.03^b^	2.36^a^	0.08	0.036
Isovalerate	4.15	4.74	0.17	0.085
Sex hormones, ng/g				
Estradiol	14.39	12.68	0.53	0.526
Progesterone	19.63^b^	50.66^a^	3.32	0.016

SEM, standard error of the mean.

Means within the same row with different superscript letters are significantly different.

1 LBW (low birth weight, *n* = 52); HBW (high birth weight, *n* = 59).

### 3.4 Correlations analysis between the microbiome and metabolome

**Figure 6:**
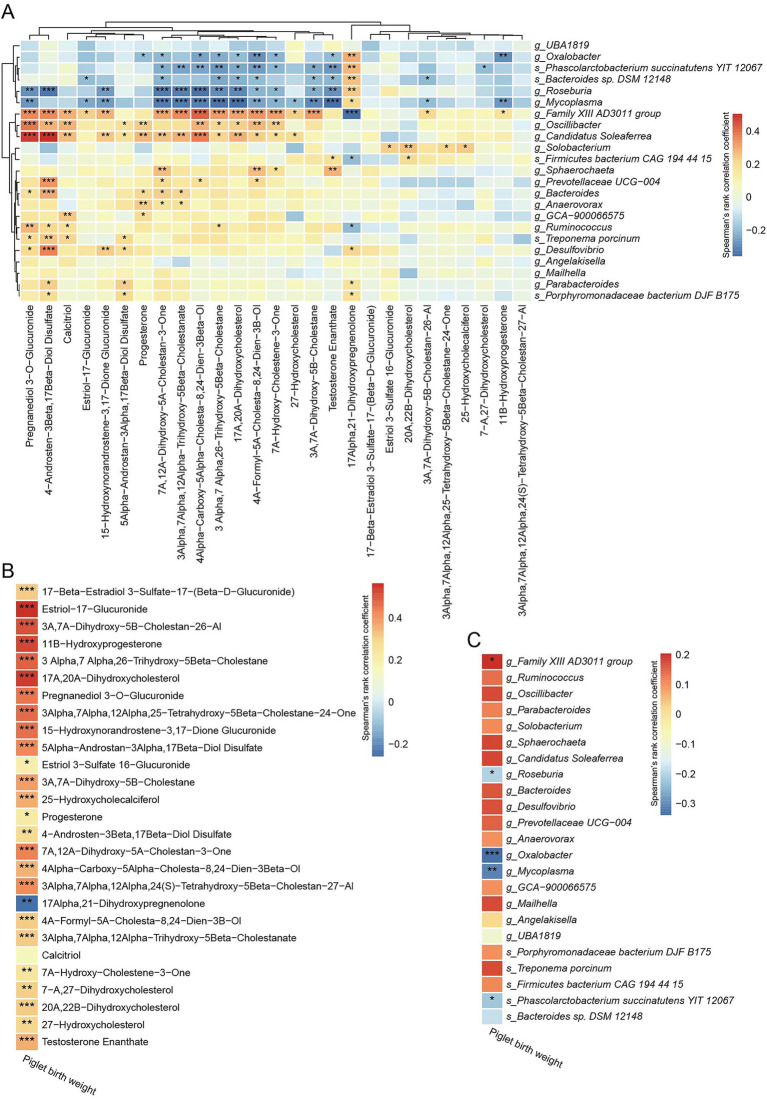
Correlations of piglet birth weight with differential gut bacterial taxa and metabolites. **(A)** Spearman correlations between significantly different bacterial taxa at the genus and species levels and metabolites related to steroids, bile acids and associated derivatives between the HBW and LBW groups. **(B)** Spearman correlations between piglet birth weight and differential metabolites related to steroids, bile acids, and associated derivatives. **(C)** Spearman correlations between piglet birth weight and differential gut bacterial taxa at the genus and species levels. In the figure, the red color represents a positive correlation, while the blue color represents the negative correlation. ^*^p < 0.05, ^**^p < 0.01, and ^***^p < 0.001.

We investigated associations between differential bacterial taxa (at the genus and species levels) and metabolites related to steroids, bile acids, and associated derivatives in the HBW and LBW groups (Figure 6A). Spearman correlation analysis showed that bacterial species significantly enriched in the HBW group were positively associated with metabolites mapped to the primary bile acid biosynthesis pathway, including 3α,7α,12α-trihydroxy-5β-cholestanate, 7α-hydroxycholestene-3-one, and 3α,7α,26-trihydroxy-5β-cholestane (Figure 6A). Consistently, several sex steroid–related metabolites, including pregnanediol 3-O-glucuronide, 4-androsten-3β,17β-diol disulfate, estriol-17-glucuronide, progesterone, and testosterone enanthate, were also positively associated with bacterial species significantly enriched in the HBW group (Figure 6A). We further investigated the associations between piglet birth weight and differential fecal metabolites related to steroids, bile acids, and associated derivatives (Figure 6B). Positive correlations were observed between piglet birth weight and the levels of several metabolites, including estriol-17-glucuronide, 3α,7α-dihydroxy-5β-cholestan-26-al, 11β-hydroxyprogesterone, 17α,20α-dihydroxycholesterol, pregnanediol 3-O-glucuronide, 3α,7α-dihydroxy-5β-cholestane, progesterone, 3α,7α,26-trihydroxy-5β-cholestane, and 7α,12α-dihydroxy-5α-cholestan-3-one (*p* < 0.05) (Figure 6B). In contrast, 17α,21-dihydroxypregnenolone was negatively correlated with piglet birth weight (*p* < 0.05) (Figure 6B). Spearman correlation analysis was performed to assess the associations between piglet birth weight and differentially abundant gut microbial taxa (Figure 6C). The abundance of the *Family XIII AD3011 group* was positively correlated with piglet birth weight (*p* < 0.05), whereas *Roseburia*, *Oxalobacter*, *Mycoplasma*, and *Phascolarctobacterium succinatutens YIT 12067* showed significant negative correlations (*p* < 0.05) (Figure 6C).

**Figure 7:**
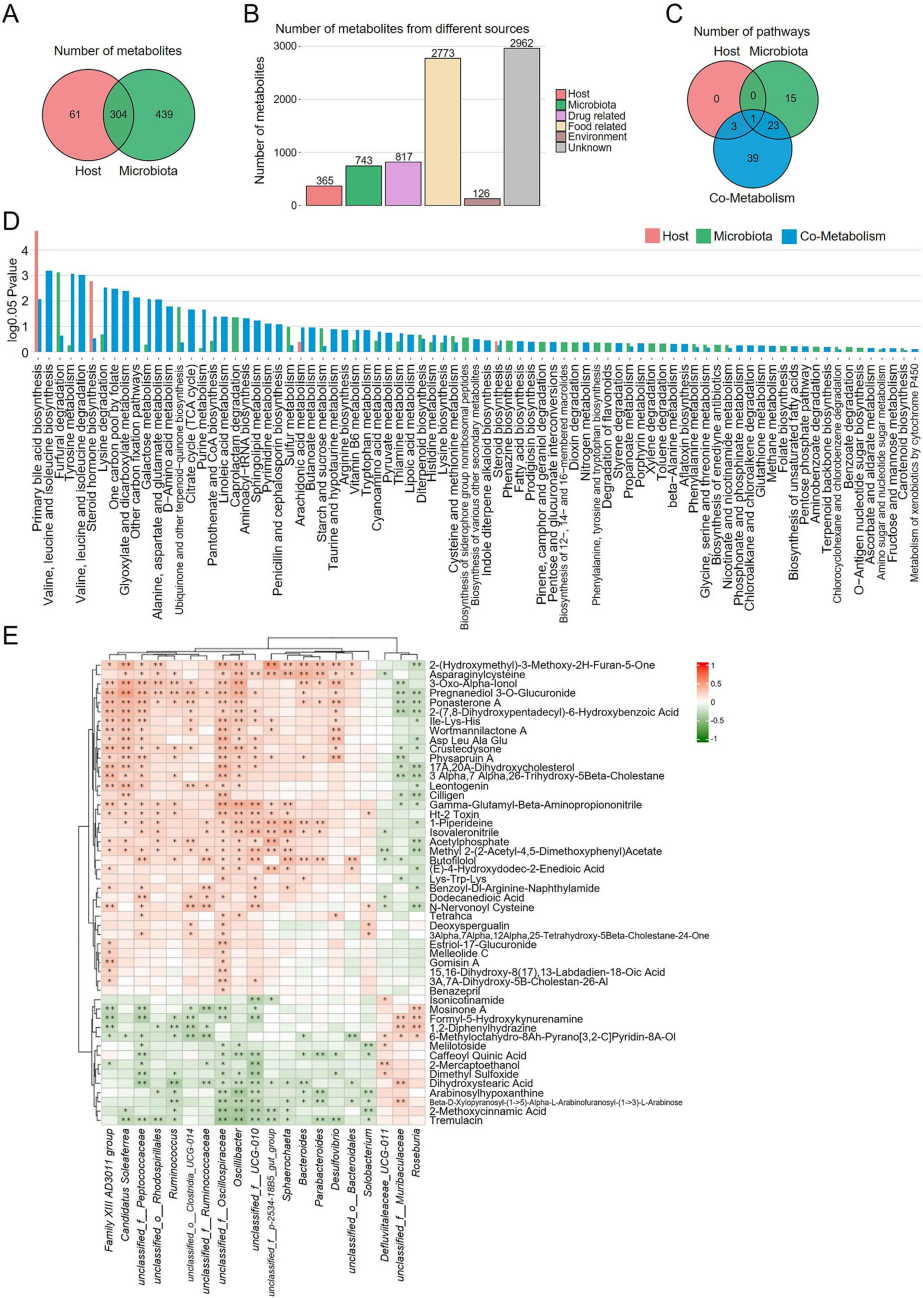
MetOrigin-based metabolic tracing of differential metabolites. **(A)** Venn diagram of differential metabolites. **(B)** Bar chart of the number of metabolites in different categories. **(C)** Venn diagram of enriched metabolic pathways. **(D)** Bar chart of enriched differential metabolic pathways. **(E)** Heatmap of Spearman correlations between the relative abundances of significantly different genera and fecal metabolites. ^*^p < 0.05 and ^**^p < 0.01.

Furthermore, the MetOrigin platform was employed to integrate statistical correlations and biological pathways linking the gut microbiome with the fecal metabolome. Classification of metabolite sources identified a total of 61 host-derived metabolites, 439 microbiota-derived metabolites, and 304 co-metabolites (Figure 7A). In addition, 817 drug-associated metabolites, 2,773 food-associated metabolites, 126 environment-associated metabolites, and 2,962 metabolites of unknown origin were identified (Figure 7B). Metabolic pathway enrichment analysis revealed that 4, 39, and 66 pathways were associated with host-specific, microbiota-specific, and co-metabolism databases, respectively (Figure 7C). Metabolic pathway enrichment analysis identified several significant pathways, including host-specific pathways such as primary bile acid biosynthesis and steroid hormone biosynthesis, microbiota-specific pathways like furfural degradation, ubiquinone and other terpenoid-quinone biosynthesis, and caprolactam degradation, with the remaining pathways primarily involving host-microbiota co-metabolism (Figure 7D). The most critical pathways included primary bile acid biosynthesis, valine, leucine, and isoleucine biosynthesis and degradation, and steroid hormone biosynthesis (Figure 7D). Spearman correlation analysis was performed between differentially abundant metabolites and bacterial genera identified in the HBW and LBW groups (Figure 7E). Among the 50 significantly different metabolites, 36 were positively correlated with bacteria enriched in the HBW group, whereas 14 showed positive correlations with bacteria enriched in the LBW group (Figure 7E).

**Figure 8:**
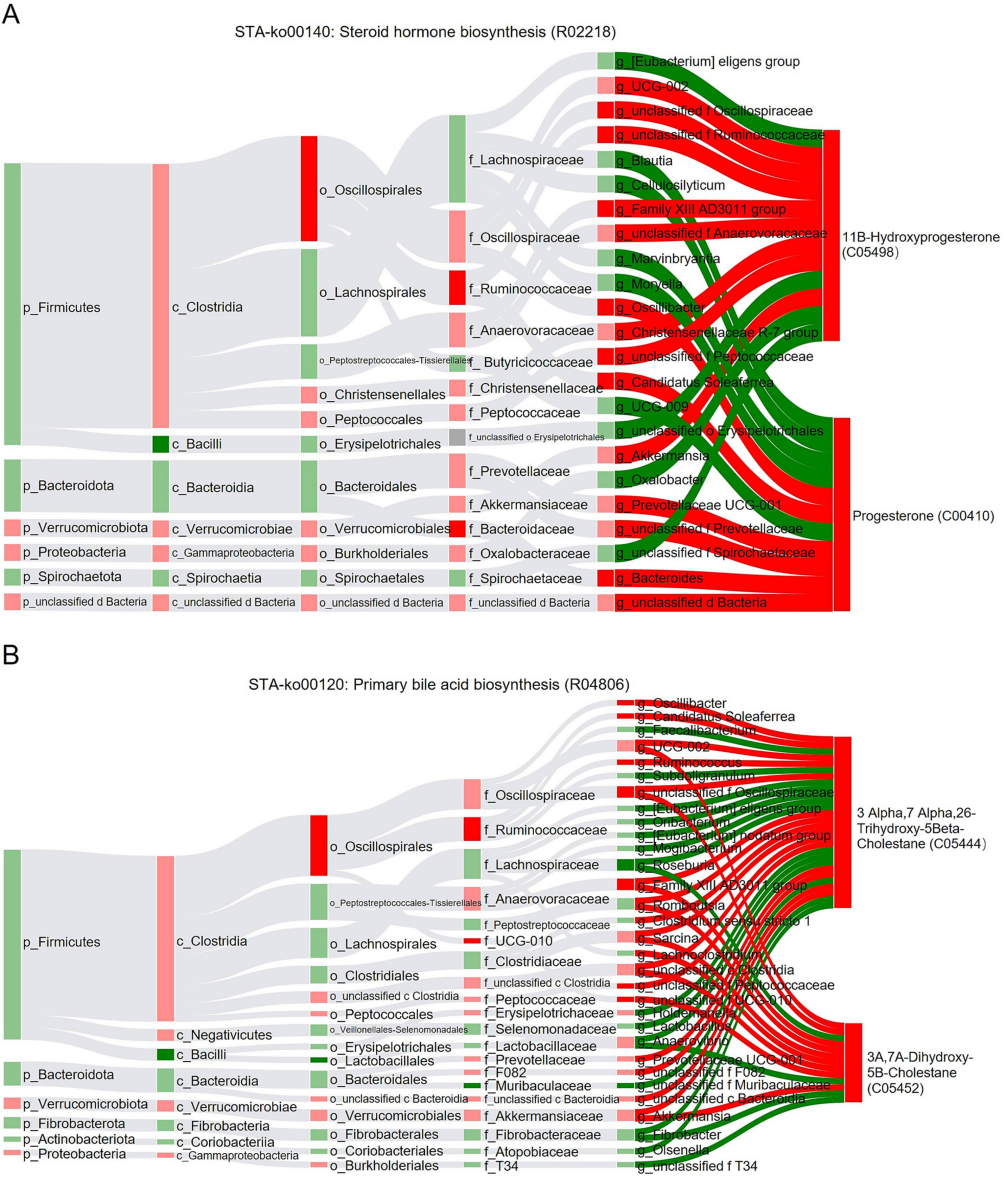
BIO-Sankey network illustrating correlations between microbes and metabolites. **(A)** The BIO-Sankey network for R02218 metabolic reaction in steroid hormone biosynthesis. **(B)** The BIO-Sankey network for R04806 metabolic reaction in primary bile acid biosynthesis. In the figure, the red color represents a positive correlation, while the blue color represents the negative correlation.

The correlations between gut microbiota at different taxonomic levels and the key reaction R02218 in the steroid hormone biosynthesis pathway were analyzed using the BIO-Sankey network on the MetOrigin platform (Figure 8A). At the order and family levels, Oscillospirales, Ruminococcaceae, and Bacteroidaceae were positively correlated with the biosynthesis of 11β-Hydroxyprogesterone and progesterone. At the genus level, the *Family XIII AD3011 group* was positively correlated with the biosynthesis of 11β-Hydroxyprogesterone, while *Oscillibacter* and *Bacteroides* were positively correlated with the biosynthesis of progesterone (Figure 8A). Similarly, correlations between gut microbiota at different taxonomic levels and the key reaction R04806 in the primary bile acid biosynthesis pathway were analyzed (Figure 8B). At the order and family levels, Oscillospirales, Ruminococcaceae, and UCG-010 were positively correlated with the biosynthesis of 3α,7α,26-trihydroxy-5β-cholestane and 3α,7α-dihydroxy-5β-cholestane; at the genus level, *Oscillibacter*, *Candidatus Soleaferrea*, *UCG-002*, *Ruminococcus*, and the *Family XIII AD3011 group* were positively correlated with the biosynthesis of 3α,7α,26-trihydroxy-5β-cholestane, while *UCG-002*, *Family XIII AD3011 group*, and *Akkermansia* were positively correlated with the biosynthesis of 3α,7α-dihydroxy-5β-cholestane (Figure 8B).

## 4 Discussion

Modern swine genotypes have been selected for increased litter size, thereby resulting in reduced average birth weight and greater within-litter birth weight variation (Quesnel et al., 2008; Langendijk et al., 2023). Improving piglet birth weight is therefore a major priority because it strongly influences preweaning survival and subsequent growth performance (Zeng et al., 2019; Ruggeri et al., 2024). In this study, we collected fecal samples from 260 sows at gestation day 100 and classified sows according to the average birth weight of live-born piglets and live litter size. For downstream multi-omics analyses, we selected two extreme groups: high birth weight (HBW; 1.41 ± 0.02 kg, 16.25 ± 0.25 piglets/litter, *n* = 59) and low birth weight (LBW; 1.07 ± 0.02 kg, 12.19 ± 0.22 piglets/litter, *n* = 52). We found that higher piglet birth weight was associated with distinct maternal gut microbiota and metabolome profiles. Compared with the LBW group, the HBW group exhibited increased SCFA-producing taxa and elevated fecal SCFA levels, along with an enhanced steroid hormone-associated metabolic profile.

HBW and LBW sows exhibited distinct gut bacterial communities, with the HBW group had significantly higher genus-level relative abundances of the *Family XIII AD3011 group*, *Ruminococcus*, *Oscillibacter*, *Parabacteroides*, *Solobacterium*, *Sphaerochaeta*, *Candidatus Soleaferrea*, *Bacteroides*, and *Desulfovibrio*. Notably, several taxa enriched in the HBW group are commonly associated with dietary fiber fermentation and SCFAs production, including *Ruminococcus*, *Bacteroides*, and *Parabacteroides* (Koh et al., 2016). Correspondingly, fecal levels of acetate, propionate, isobutyrate, and valerate were significantly higher in the HBW group. SCFAs have been implicated in metabolic and immune regulation during pregnancy, with evidence that they attenuate inflammatory signaling in maternal tissues and the placenta and thereby may support placental function and fetal development (Roy et al., 2020; Pronovost et al., 2023; Qin et al., 2025). Mechanistically, in an intrauterine growth restriction (IUGR) mouse model, enrichment of *Lactobacillus murinus* was accompanied by higher luminal and circulating SCFA levels, which have been reported to improve placental function by alleviating placental oxidative stress and inflammation, thereby enhancing placental efficiency and supporting fetal growth (Feng et al., 2025). Furthermore, dietary supplementation with sodium butyrate or sodium acetate during gestation has been reported to improve reproductive outcomes, as reflected by increased piglet birth weight and a reduced proportion of low-viability piglets, respectively (Zhao et al., 2021; Qi et al., 2023), suggesting that higher maternal gut SCFA levels are associated with improved fetal growth. We also observed enrichment of *Oscillibacter* in HBW sows. *Oscillibacter valericigenes* can produce valerate (Iino et al., 2007) and has been associated with intestinal barrier function and host cholesterol-related metabolic pathways, including bile acid and steroid metabolism (Hu et al., 2024; Li C. et al., 2024). In line with these reports, valerate and multiple bile acid- and steroid-related metabolites were elevated in the HBW group, raising the possibility that *Oscillibacter* enrichment may be associated with coordinated shifts in microbial SCFA output and host bile acid and steroid metabolic profiles during late gestation.

Beyond SCFA-related metabolism, our fecal metabolomic profiling further identified enrichment of primary bile acid and steroid hormone biosynthesis pathways in HBW sows. In mammals, cholesterol serves as the precursor for the biosynthesis of both bile acids and steroid hormones (Cui et al., 2025). Our results showed increased levels of multiple cholesterol-derived metabolites in the HBW group, including 3α,7α-dihydroxy-5β-cholestan-26-al, 3α,7α,12α-trihydroxy-5β-cholestanate, 7α-hydroxy-cholestene-3-one, 7α,27-dihydroxycholesterol, 27-hydroxycholesterol, 3α,7α-dihydroxy-5β-cholestane, and 3α,7α,26-trihydroxy-5β-cholestane, suggesting an altered cholesterol-derived metabolic profile that is closely related to primary bile acid biosynthesis. This pattern is consistent with the enrichment of *Oscillibacter* in the HBW group, a genus previously linked to cholesterol metabolism, and supports an association between *Oscillibacter* abundance and sterol-related metabolic features (Li C. et al., 2024). Bile acid metabolism plays an important regulatory role in host sex-hormone metabolism and offspring birth weight in sows. For example, chenodeoxycholic acid administration during early gestation improved embryo implantation in sows and was accompanied by increased maternal serum progesterone, as well as modulation of gut microbiota and bile acid homeostasis (Chen M. et al., 2023). Conversely, gestational bile acid dysregulation, often reflected by elevated serum total bile acids, has been linked to impaired placental development, potentially compromising nutrient transfer and increasing the risk of low birth weight and IUGR (Wang et al., 2019; Song et al., 2021). Consistently, gestational soluble fiber supplementation increased piglet birth weight and reduced the proportion of IUGR piglets, and these benefits were accompanied by improved bile acid homeostasis, shifts in bile acid-related microbial taxa, and altered fecal bile acid profiles (Wu et al., 2021). Similarly, galacto-oligosaccharides combined with hyocholic acids increased piglet birth weight and was accompanied by enhanced antioxidant capacity and modulation of gut microbiota during late gestation (Yu J. et al., 2024). Taken together, these findings highlight bile acid metabolism as a potential microbiota–host axis associated with sow endocrine status and offspring birth weight. However, whether the HBW-associated increase in primary bile acid-related metabolites primarily reflects altered bile acid homeostasis or bile acid signaling that impacts maternal steroid hormone balance remains to be determined.

Progesterone is essential for pregnancy maintenance and placental function (Mesiano, 2001). We observed higher fecal levels of progesterone and its metabolites (e.g., 11β-hydroxyprogesterone and pregnanediol 3-O-glucuronide) in HBW sows; this observation aligns with reports that progesterone supplementation can reduce early farrowing and increased piglet birth weight (Gaggini et al., 2013). We also observed increased levels of multiple sulfated and glucuronidated androgens and estrogens (e.g., 15-hydroxynorandrostene-3,17-dione glucuronide, 5α-Androstan-3α,17β-diol disulfate, estriol-17-glucuronide, estriol 3-sulfate 16-glucuronide, and 17-β-estradiol 3-sulfate-17-(β-d-glucuronide) in HBW sows). These metabolites can be deconjugated by gut microbial enzymes, such as β-glucuronidase, which facilitates the reabsorption of free estriol via enterohepatic circulation and thereby contributes to the regulation of systemic estrogen levels during late pregnancy (Pace and Watnick, 2021). Gut microbiota and its derived metabolites are associated with the endocrine and reproductive systems (Xu et al., 2021; Xu et al., 2023). For example, an integrated analysis has provided evidence that *Lactobacillus reuteri* and *Prevotella* spp. participate in the degradation of pregnenolone, progesterone, and testosterone, thereby promoting estrogen biosynthesis and facilitating the normal transition from weaning to estrus in sows (Liu et al., 2023). In our dataset, HBW-enriched taxa were positively associated with primary bile acids and multiple sex hormone-related metabolites, and these metabolite features were also positively correlated with piglet birth weight. Overall, metabolomic profiling revealed more pronounced estrogen-, progesterone-, and androgen-related metabolic profiles in the HBW group than in the LBW group, suggesting a positive association between enhanced sex hormone-related metabolism and higher piglet birth weight. To strengthen interpretability and source attribution of the metabolomic profiles, we integrated microbiome-metabolome data using the MetOrigin platform (Yu G. et al., 2024), which classifies metabolites by biological origin (e.g., host-, microbiome-, and diet-derived). This source-guided analysis suggested that the group differences were driven by both host and microbial activities, highlighting primary bile acid and steroid hormone biosynthesis as key pathways. The integrated Sankey network further indicated positive associations between HBW-enriched taxa and progesterone and bile acid metabolism. However, although fecal steroid hormone metabolites represent time-integrated outputs and can reflect maternal endocrine status, their relevance to systemic endocrine regulation in sows remains to be established and warrants further investigation.

While our work provides the gut microbial composition and metabolite profiles of sows with high versus low piglet birth weights, several limitations should be noted. First, the maternal gut microbiome undergoes substantial changes across gestation; therefore, because our data were collected at a single late-gestation time point (gestation day 100), it remains unclear whether the microbiome-birth weight associations observed here emerge during mid-to-late gestation and persist into late gestation or instead represent a late-gestation adaptive response driven by increased maternal nutrient demands and host-microbiome interactions. Second, because comprehensive placental phenotyping was not available, mechanistic interpretation linking microbial and metabolic differences to fetal growth remains limited. Future longitudinal sampling from mid- to late-gestation, combined with detailed placental phenotyping (e.g., placental weight, vascularization, and nutrient transport markers), together with targeted nutritional and microbiome interventions, will be essential to validate candidate taxa and metabolites and to clarify their potential links to fetal growth and offspring birth weight.

## 5 Conclusion

In conclusion, we observed clear differences in gut microbiota composition and fecal metabolic profiles between sows in the HBW and LBW groups. Integrated analyses suggest coordinated positive associations among HBW-enriched microbial taxa, increased SCFAs, and enhanced primary bile acid and steroid hormone-related metabolic features, including progesterone, which together align with higher piglet birth weight. Given the cross-sectional nature of this study, these relationships should be interpreted as associations rather than evidence of causality. Nevertheless, our findings expand current understanding of maternal gut microbe-metabolite networks during late gestation and their potential links to fetal growth, and support the possibility that microbiome-informed nutritional or microbial interventions may be a promising avenue to improve piglet birth weight.

## Data Availability

The sequencing data from this study are available in the National Center for Biotechnology Information (NCBI) SRA repository under accession numbers PRJNA1226750. The untargeted metabolomic data have been deposited in the EMBL-EBI MetaboLights database under the identifier MTBLS13791.

## Footnotes

1
https://cloud.majorbio.com

## References

[ref1] Almeida F. Alvarenga Dias A. Moreira L. P. Fiúza A. Chiarini-Garcia H. (2017). Ovarian follicle development and genital tract characteristics in different birthweight gilts at 150 days of age. Reprod. Domest. Anim. 52, 756–762. doi: 10.1111/rda.12976, 28432701

[ref2] Alves K. Schenkel F. S. Brito L. F. Robinson A. (2018). Estimation of direct and maternal genetic parameters for individual birth weight, weaning weight, and probe weight in Yorkshire and landrace pigs. J. Anim. Sci. 96, 2567–2578. doi: 10.1093/jas/sky172, 29762734 PMC6095450

[ref3] Boonkum W. Permthongchoochai S. Chankitisakul V. Duangjinda M. (2025). Genetic strategies for enhancing litter size and birth weight uniformity in piglets. Front. Vet. Sci. 12:1512701. doi: 10.3389/fvets.2025.1512701, 40196805 PMC11973344

[ref4] Chen Y. Ou Z. Pang M. Tao Z. Zheng X. Huang Z. . (2023). Extracellular vesicles derived from *Akkermansia muciniphila* promote placentation and mitigate preeclampsia in a mouse model. J. Extracell. Vesicles 12:e12328. doi: 10.1002/jev2.12328, 37165987 PMC10173384

[ref5] Chen Z. Yang H. Fu H. Wu L. Liu M. Jiang H. . (2022). Gut bacterial species in late trimester of pregnant sows influence the occurrence of stillborn piglet through pro-inflammation response. Front. Immunol. 13:1101130. doi: 10.3389/fimmu.2022.1101130, 36741405 PMC9890068

[ref6] Chen M. Zhao Y. Ji H. Li L. Liu H. Wang S. . (2023). Chenodeoxycholic acid improves embryo implantation and metabolic health through modulating gut microbiota-host metabolites interaction during early pregnancy. Antioxidants 13:8. doi: 10.3390/antiox13010008, 38275628 PMC10812749

[ref7] China Feed Database. (2020). Tables of feed composition and nutritive values in China (in Chinese). Available at: https://www.chinafeeddata.org.cn/admin/Login/slcfb (Accessed February 7, 2025).

[ref8] Coskun R. Chang Z. L. Marcial Rodríguez A. Liu H. Cheng J. Alippe Y. . (2025). Effects of the gut microbiota on placental angiogenesis and intrauterine growth in gnotobiotic mice. Proc. Natl. Acad. Sci. U.S.A. 122:e2426341122. doi: 10.1073/pnas.2426341122, 40711921 PMC12318179

[ref9] Cui D. Yu X. Guan Q. Shen Y. Liao J. Liu Y. . (2025). Cholesterol metabolism: molecular mechanisms, biological functions, diseases, and therapeutic targets. Mol. Biomed. 6:72. doi: 10.1186/s43556-025-00321-3, 41062796 PMC12508344

[ref10] Declerck I. Sarrazin S. Dewulf J. Maes D. (2017). Sow and piglet factors determining variation of colostrum intake between and within litters. Animal 11, 1336–1343. doi: 10.1017/s1751731117000131, 28193309

[ref11] Fang T. Tian G. Chen D. He J. Zheng P. Mao X. . (2024). Endoplasmic reticulum stress contributes to intestinal injury in intrauterine growth restriction newborn piglets. Animals 14:2677. doi: 10.3390/ani14182677, 39335266 PMC11429086

[ref12] Feng C. Wu Y. Zhang X. Wang S. Wang J. Yang H. (2025). Maternal milk fat globule membrane enriched gut *L. murinus* and circulating SCFAs to improve placental efficiency and fetal development in intrauterine growth restricted mice model. Gut Microbes 17:2449095. doi: 10.1080/19490976.2024.2449095, 39762283 PMC12931695

[ref13] Gaggini T. S. Perin J. Arend L. S. Bernardi M. L. Wentz I. Bortolozzo F. P. (2013). Altrenogest treatment associated with a farrowing induction protocol to avoid early parturition in sows. Reprod. Domest. Anim. 48, 390–395. doi: 10.1111/rda.12085, 22994857

[ref14] Gondret F. Lefaucheur L. Juin H. Louveau I. Lebret B. (2006). Low birth weight is associated with enlarged muscle fiber area and impaired meat tenderness of the longissimus muscle in pigs. J. Anim. Sci. 84, 93–103. doi: 10.2527/2006.84193x, 16361495

[ref15] Han L. Azad M. A. K. Huang P. Wang W. Zhang W. Blachier F. . (2022). Maternal supplementation with different probiotic mixture from late pregnancy to day 21 postpartum: consequences for litter size, plasma and colostrum parameters, and fecal microbiota and metabolites in sows. Front. Vet. Sci. 9:726276. doi: 10.3389/fvets.2022.726276, 35211537 PMC8860973

[ref16] Hu J. Chen J. Ma L. Hou Q. Zhang Y. Kong X. . (2024). Characterizing core microbiota and regulatory functions of the pig gut microbiome. ISME J. 18:wrad037. doi: 10.1093/ismejo/wrad037, 38366194 PMC10873858

[ref17] Huang S. Wu D. Hao X. Nie J. Huang Z. Ma S. . (2023). Dietary fiber supplementation during the last 50 days of gestation improves the farrowing performance of gilts by modulating insulin sensitivity, gut microbiota, and placental function. J. Anim. Sci. 101:skad021. doi: 10.1093/jas/skad021, 36634095 PMC9912709

[ref18] Iino T. Mori K. Tanaka K. Suzuki K. I. Harayama S. (2007). *Oscillibacter valericigenes* gen. nov., sp. nov., a valerate-producing anaerobic bacterium isolated from the alimentary canal of a Japanese corbicula clam. Int. J. Syst. Evol. Microbiol. 57, 1840–1845. doi: 10.1099/ijs.0.64717-0, 17684268

[ref19] Kim S. W. Weaver A. C. Shen Y. B. Zhao Y. (2013). Improving efficiency of sow productivity: nutrition and health. J. Anim. Sci. Biotechnol. 4:26. doi: 10.1186/2049-1891-4-26, 23885840 PMC3733949

[ref20] Koh A. De Vadder F. Kovatcheva-Datchary P. Bäckhed F. (2016). From dietary fiber to host physiology: short-chain fatty acids as key bacterial metabolites. Cell 165, 1332–1345. doi: 10.1016/j.cell.2016.05.041, 27259147

[ref21] Langendijk P. Fleuren M. Page G. (2023). Review: targeted nutrition in gestating sows: opportunities to enhance sow performance and piglet vitality. Animal 17:100756. doi: 10.1016/j.animal.2023.100756 36967294

[ref22] Li N. Huang S. Jiang L. Wang W. Li T. Zuo B. . (2018). Differences in the gut microbiota establishment and metabolome characteristics between low- and normal-birth-weight piglets during early-life. Front. Microbiol. 9:1798. doi: 10.3389/fmicb.2018.01798, 30245669 PMC6137259

[ref23] Li S. Lu T. Lin Z. Zhang Y. Zhou X. Li M. . (2024). Supplementation with probiotics co-cultivation improves the reproductive performance in a sow-piglet model by mother-infant microbiota transmission and placental mTOR signaling. World J. Microbiol. Biotechnol. 41:13. doi: 10.1007/s11274-024-04222-5 39704872

[ref24] Li C. Stražar M. Mohamed A. M. T. Pacheco J. A. Walker R. L. Lebar T. . (2024). Gut microbiome and metabolome profiling in Framingham heart study reveals cholesterol-metabolizing bacteria. Cell 187, 1834–1852.e19. doi: 10.1016/j.cell.2024.03.014, 38569543 PMC11071153

[ref25] Li Y. Yang M. Zhang L. Mao Z. Lin Y. Xu S. . (2022). Dietary fiber supplementation in gestating sow diet improved fetal growth and placental development and function through serotonin signaling pathway. Front. Vet. Sci. 9:831703. doi: 10.3389/fvets.2022.831703, 35647096 PMC9133666

[ref26] Liu M. Zhang J. Zhou Y. Xiong S. Zhou M. Wu L. . (2023). Gut microbiota affects the estrus return of sows by regulating the metabolism of sex steroid hormones. J. Anim. Sci. Biotechnol. 14:155. doi: 10.1186/s40104-023-00959-5, 38115159 PMC10731813

[ref27] Ma Z. Wu Z. Wang Y. Meng Q. Chen P. Li J. . (2023). Effect of yeast culture on reproductive performance, gut microbiota, and milk composition in primiparous sows. Animals 13:2954. doi: 10.3390/ani13182954, 37760354 PMC10525930

[ref28] Mbuthia J. M. Kasper C. Zenk M. Bee G. Metges C. C. Daş G. (2025). Predicting piglet survival until weaning using birth weight and within-litter birth weight variation as easily measured proxy predictors. Animal 19:101479. doi: 10.1016/j.animal.2025.101479, 40154104

[ref29] Mcpherson R. L. Ji F. Wu G. Blanton J. R. Jr. Kim S. W. (2004). Growth and compositional changes of fetal tissues in pigs. J. Anim. Sci. 82, 2534–2540. doi: 10.2527/2004.8292534x, 15446468

[ref30] Mesiano S. (2001). Roles of estrogen and progesterone in human parturition. Front. Horm. Res. 27, 86–104. doi: 10.1159/000061038 11450438

[ref31] Pace F. Watnick P. I. (2021). The interplay of sex steroids, the immune response, and the intestinal microbiota. Trends Microbiol. 29, 849–859. doi: 10.1016/j.tim.2020.11.001, 33257138 PMC12358074

[ref32] Pronovost G. N. Yu K. B. Coley-O'rourke E. J. L. Telang S. S. Chen A. S. Vuong H. E. . (2023). The maternal microbiome promotes placental development in mice. Sci. Adv. 9:eadk1887. doi: 10.1126/sciadv.adk1887, 37801498 37801498 PMC10558122

[ref33] Qi Y. Zheng T. Yang S. Zhang Q. Li B. Zeng X. . (2023). Maternal sodium acetate supplementation promotes lactation performance of sows and their offspring growth performance. Anim. Nutr. 14, 213–224. doi: 10.1016/j.aninu.2023.04.003, 37484994 PMC10362078

[ref34] Qin X. Zhang M. Chen S. Tang Y. Cui J. Ding G. (2025). Short-chain fatty acids in fetal development and metabolism. Trends Mol. Med. 31, 625–639. doi: 10.1016/j.molmed.2024.11.014, 39694776

[ref35] Quesnel H. Brossard L. Valancogne A. Quiniou N. (2008). Influence of some sow characteristics on within-litter variation of piglet birth weight. Animal 2, 1842–1849. doi: 10.1017/s175173110800308x, 22444091

[ref36] Roy R. Nguyen-Ngo C. Lappas M. (2020). Short-chain fatty acids as novel therapeutics for gestational diabetes. J. Mol. Endocrinol. 65, 21–34. doi: 10.1530/jme-20-0094, 32580157

[ref37] Ruggeri R. Bee G. Trevisi P. Ollagnier C. (2024). Intrauterine growth restriction defined by increased brain-to-liver weight ratio affects postnatal growth and protein efficiency in pigs. Animal 18:101044. doi: 10.1016/j.animal.2023.101044, 38128172

[ref38] Song F. Chen Y. Chen L. Li H. Cheng X. Wu W. (2021). Association of elevated maternal serum total bile acids with low birth weight and intrauterine fetal growth restriction. JAMA Netw. Open 4:e2117409. doi: 10.1001/jamanetworkopen.2021.17409, 34279647 PMC8290304

[ref39] Tu X. Duan C. Lin B. Li K. Gao J. Yan H. . (2022). Characteristics of the gut microbiota in pregnant women with fetal growth restriction. BMC Pregnancy Childbirth 22:297. doi: 10.1186/s12884-022-04635-w, 35392848 PMC8991653

[ref40] Wang P. Zhong H. Song Y. Yuan P. Li Y. Lin S. . (2019). Targeted metabolomics analysis of maternal-placental-fetal metabolism in pregnant swine reveals links in fetal bile acid homeostasis and sulfation capacity. Am. J. Physiol. Gastrointest. Liver Physiol. 317, G8–G16. doi: 10.1152/ajpgi.00056.2019, 31021171

[ref41] Want E. J. Masson P. Michopoulos F. Wilson I. D. Theodoridis G. Plumb R. S. . (2013). Global metabolic profiling of animal and human tissues via UPLC-MS. Nat. Protoc. 8, 17–32. doi: 10.1038/nprot.2012.135, 23222455

[ref42] Wu X. Yin S. Cheng C. Xu C. Peng J. (2021). Inclusion of soluble fiber during gestation regulates gut microbiota, improves bile acid homeostasis, and enhances the reproductive performance of sows. Front. Vet. Sci. 8:756910. doi: 10.3389/fvets.2021.756910, 34869730 PMC8635514

[ref43] Xu B. Qin W. Chen Y. Tang Y. Zhou S. Huang J. . (2023). Multi-omics analysis reveals gut microbiota-ovary axis contributed to the follicular development difference between Meishan and Landrace × Yorkshire sows. J. Anim. Sci. Biotechnol. 14:68. doi: 10.1186/s40104-023-00865-w, 37122038 PMC10150527

[ref44] Xu B. Qin W. Yan Y. Tang Y. Zhou S. Huang J. . (2021). Gut microbiota contributes to the development of endometrial glands in gilts during the ovary-dependent period. J. Anim. Sci. Biotechnol. 12:57. doi: 10.1186/s40104-021-00578-y, 33947457 PMC8097987

[ref45] Ye Q. Luo T. Han L. Chen Y. Hu Y. Jiang H. . (2024). Multi-omics analysis reveals the dominant intestinal microbial strains and metabolites related to the reproductive performance in pregnant sows. Anim. Nutriomics 1:e5. doi: 10.1017/anr.2024.7

[ref46] Yu J. Wang J. Cao C. Gong J. Cao J. Yin J. . (2024). Maternal intervention with a combination of galacto-oligosaccharides and hyocholic acids during late gestation and lactation increased the reproductive performance, colostrum composition, antioxidant and altered intestinal microflora in sows. Front. Microbiol. 15:1367877. doi: 10.3389/fmicb.2024.1367877, 38933026 PMC11199897

[ref47] Yu G. Xu C. Wang X. Ju F. Fu J. Ni Y. (2024). Metorigin 2.0: advancing the discovery of microbial metabolites and their origins. iMeta 3:e246. doi: 10.1002/imt2.246, 39742299 PMC11683456

[ref48] Zeng Z. K. Urriola P. E. Dunkelberger J. R. Eggert J. M. Vogelzang R. Shurson G. C. . (2019). Implications of early-life indicators for survival rate, subsequent growth performance, and carcass characteristics of commercial pigs1. J. Anim. Sci. 97, 3313–3325. doi: 10.1093/jas/skz223, 31257437 PMC6667258

[ref49] Zhao Y. Wang D. Huang Y. Wu D. Ji X. Zhou X. . (2021). Maternal butyrate supplementation affects the lipid metabolism and fatty acid composition in the skeletal muscle of offspring piglets. Anim. Nutr. 7, 959–966. doi: 10.1016/j.aninu.2020.11.017, 34703913 PMC8521173

